# Supplementary material for: Discovery of bilaterian-type through-guts in cloudinomorphs from the terminal Ediacaran Period
Source: Nat Commun. 2020 Jan 10;11:205. doi: 10.1038/s41467-019-13882-z (PMC6954273; doi:10.1038/s41467-019-13882-z)
Supplement: Supplementary file 1 — Supplementary Information [file 41467_2019_13882_MOESM1_ESM.pdf]

Supplementary Information

Discovery of bilaterian-type through-guts in cloudinomorphs from the terminal Ediacaran Period

James D. Schiffbauer[1,2]*, Tara Selly[2,1]*, Sarah M. Jacquet[1], Rachel A. Merz[3], Lyle L. Nelson[4], Michael A. Strange[5], Yaoping Cai[6], Emily F. Smith[4].

[1]Department of Geological Sciences, University of Missouri, Columbia MO 65211, USA.

[2]X-ray Microanalysis Core, University of Missouri, Columbia MO 65211, USA.

[3]Biology Department, Swarthmore College, Swarthmore PA 19081, USA.

[4]Department of Earth and Planetary Sciences, Johns Hopkins University, Baltimore, Maryland 21218, USA.

[5]Department of Geoscience, University of Nevada, Las Vegas, Las Vegas NV 89154, USA.

[6]Shaanxi Key Laboratory of Early Life and Environment, State Key Laboratory of Continental Dynamics, and Department of Geology, Northwest University, Xi'an 710069, China.

*Correspondence to: schiffbauerj@missouri.edu and sellyt@missouri.edu.

This pdf file contains:

- Supplementary Discussion
- Supplementary Figures 1–3
- Supplementary Table 1
- Supplementary References

Supplementary Discussion

Hemichordates

Although not a common interpretation for cloudinomorphs, the tubicolous and vermiform pterobranch hemichordates do show some tubular similarities with organic-walled representatives of the morphoclade and thus have been previously considered[1]. The robust tubes of the pterobranchs have left a considerable fossil record extending to the early Cambrian. They have additionally shown soft-tissue preservation, with a single example from the Chengjiang Lagerstätte[2]. While no taphonomic details were reported, these soft tissues are presumed to have been pyritized but compressed (e.g., two-dimensionally pyritized, as based on observation of the published plate)[2]. Commonly colonial, the pterobranchs are stalked zooids with U-shaped guts that live within collared tubes (Supplementary Fig. 1a). Although their digestive tract may not fit with the cylindrical morphology observed here, their contractile stalks[3], on the other hand, may be a feasible non-gut interpretation—comparable in shape, position within the external tube, and with lengths that can extend through the entirety of the external tube. Pterobranch stalks, however, are densely muscular structures with a ventral nerve[3], and thus are reasonably difficult to reconcile with the sediment-infilled portions of the cylinders as observed here.

It may also be appropriate to consider the sister class to the pterobranchs, enteropneust hemichordates. The acorn worms are not tube-builders in the modern-day; although, with a few Cambrian tubicolous representatives, perhaps this was a more common life mode early in their evolutionary history[4,5]. For instance, the well-known Burgess Shale fossil, *Margaretia dorus*, originally assigned to green algae, has recently been shown to be a tubular dwelling structure of the vermiform enteropneust *Oesia disjuncta*[5]. As opposed to the U-shaped gut of the pterobranchs, the acorn worms have an anterior mouth and posterior anus connected by a straight through-gut (Supplementary Fig. 1b), which has been preserved in Cambrian representatives[4]. These are decidedly more comparable to the cylinders reported here, and the lack of hepatic sacs would suggest an affinity with modern harrimaniid worms, also similar to Cambrian representatives[4]. Their external tubes, however, may pose the most significant obstacle for such an assignment of the cloudinomorphs. The reported Cambrian tubes are solely organic in composition and their construction can be quite distinct, like the ornately perforated and anteriorly enclosed tube of *Oesia*[5].

Phoronids and stem-lophotrochozoans

Limited Cambrian examples of possible phoronids have been reported[6,7]. Much like the broader group cloudinomorphs, these horseshoe worm fossils exhibited both ‘soft shell’[6] and mineralized[7] tubes, with calcareous tubes suggested as ancestral[8]—counter to previous inferences regarding possible ancestral relationships in the cloudinomorphs[9,10]. Phoronids may also serve as the most reasonable extant analogue to the extinct worm-like tentaculitids[8], including the microconchids, which have been offered as a potential interpretation for the cloudinomorphs[11]. Possible tabulae in *Cloudina* from Spain[11] have been utilized to support the microconchid reconstruction, but these structures are tenuous within a heavily recrystallized, sparry calcite-replaced specimen and thus may not be the most biologically informative of features. With regard to their internal anatomy, phoronids and microconchids, as other

lophophorates, should have a digestive tract that follows a U-shaped path with a superiorly positioned mouth and anus (Supplementary Fig. 1c), again distinct from the morphology of the straight cylinders observed here.

While a straight through-gut has typically been considered homologous in bilaterians, recent discussion on lophotrochozoan anatomical organization and evolution[12] proposes instead that, in sessile forms, U-shaped guts may be the basal groundplan. This claim has roots in the Cambrian fossil record, with fossil U-shaped guts documented for instance in stem-rhynchonelliform brachiopods[13] and orthothecimorph hyoliths[14]. The 'U-tube theory' could imply that the cloudinomorphs, if stem-lophotrochozoans, would be expected to follow suit. There are, however, several caveats that may argue against this idea[12]. Perhaps the most important of these stipulations is that not all sessile tube-dwellers possess a U-shaped gut. For instance, some Cambrian organisms like the problematic *Hyolithellus* have been inferred to possess a straight through-gut and are interpreted to be most likely annelid-grade, potentially similar to chaetopterid polychaetes[15]. If indeed guts, the soft-tissue structures observed here show no evidence of following a U-shaped path, which may call into question either the 'U-tube theory' on ancestral U-shaped guts or the suggestion of a basal lophotrochozoan position for the cloudinomorphs[12] (Supplementary Fig. 3).

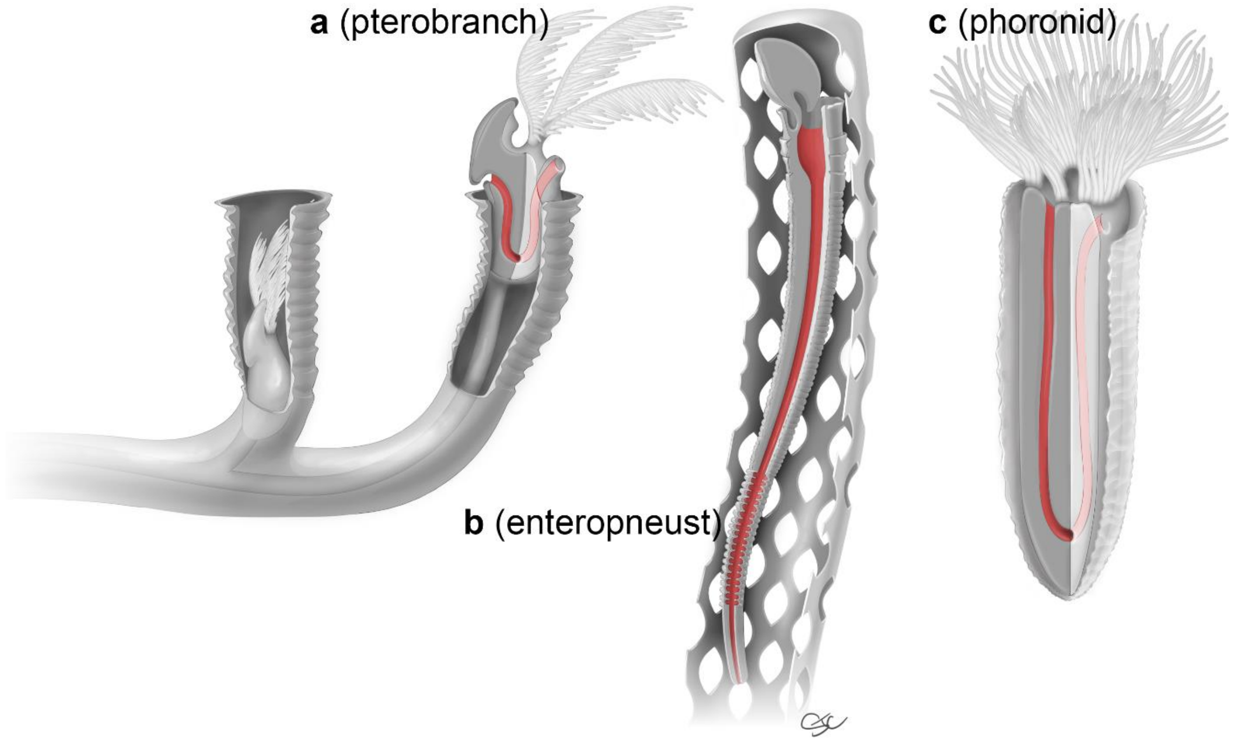

Supplementary Fig. 1. (a) Colonial pterobranch hemichordates; zooid on right illustrates U-shaped gut path. Contractile stalk shown in tube cut-out below zooid on right, and contracted zooid shown on left. (b) Stylized Cambrian enteropneust[5], showing *Margaretia*-like tube structure and straight through-gut path and reduced hepatic sacs. (c) Phoronid with deep U-shaped gut path. Illustration by Stacy Turpin Cheavens.

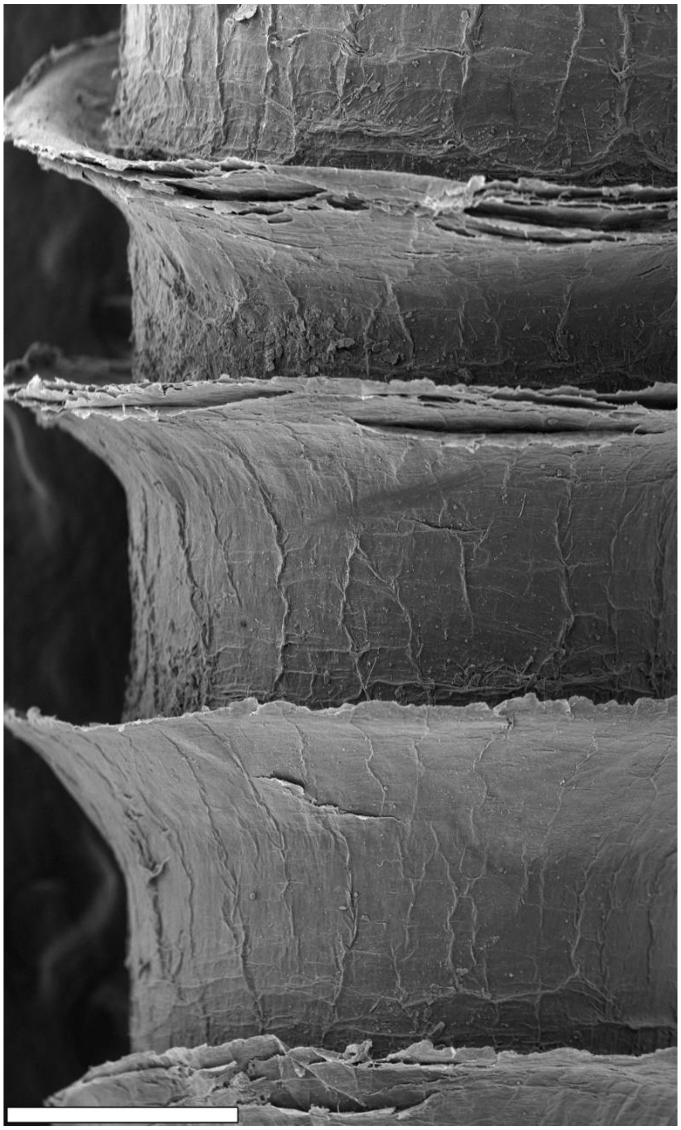

Supplementary Fig. 2. SEM micrograph of exterior tube structure of *Oasisia alvinae*, a modern funnel-in-funnel tube-building siboglinid polychaete. Scale = 500 µm.

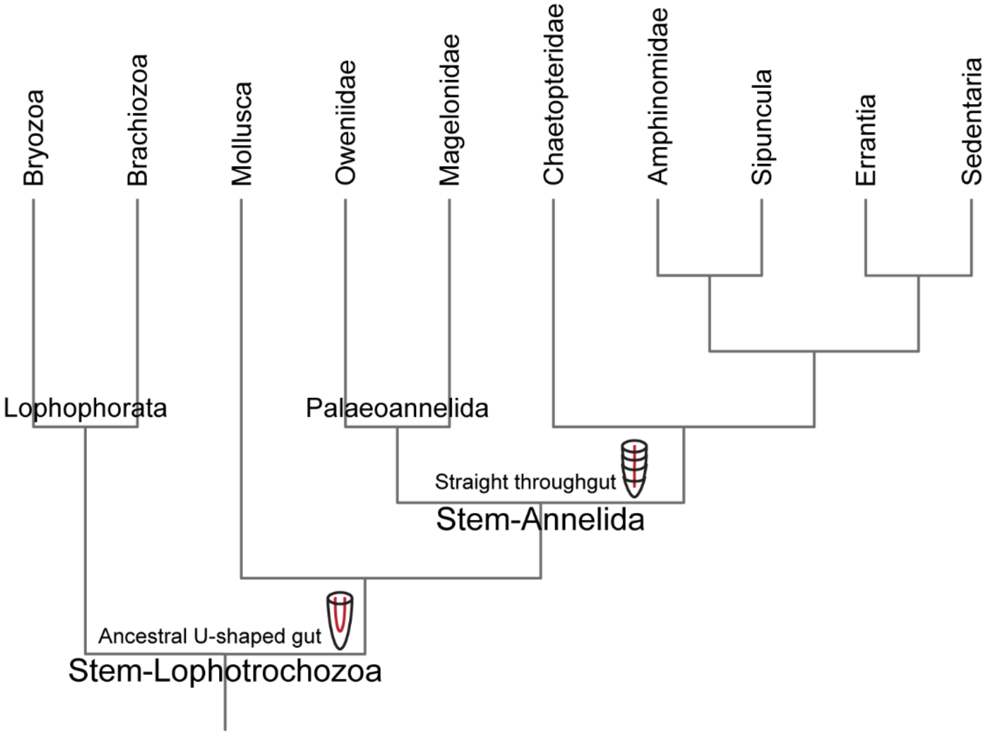

Supplementary Fig. 3. Generalized phylogenetic scenario for divergence of stem-Lophotrochozoa and stem-Annelida with gut shape noted after refs.[12,16].

Supplementary Table 1. [next page] Comparative morphological characteristics of fossil and modern tube-dwelling organisms.

		Cloudinomorphs	Anthozoans	Pterobranchs	Enteropneusts	Phoronids	Polychaetes
Modern or fossil		Fossil	Both	Both	Tube-dwellers only known from fossils	Both	Both
External tube	External tube composition	Calcareous and/or undetermined organic	Calcareous (hexacorallians) and/or mucoid organic (ceriantharians and octocorallians)	Organic, proteinaceous and polysaccharides	Undetermined organic	Organic, chitinous	Calcareous (serpulids, sabellids, cirratulids) and/or organic, proteinaceous and polysaccharides
	External shape	Nested, collared funnels	Can be simple tubular, but variable	Tubular, collared	Tubular; can be perforated or helicoidal, collared?	Wrinkled, paper-thin	Tubular, some with repeating collared units (e.g., some siboglinids and chaetopterids)
	Inner skeletal wall	Smooth	Possess septa	Smooth	No information	Smooth	Smooth
	Granular microstructure	Reported in *Cloudina*	No	No	No	No	No
	Closed posterior base	Reported in *Cloudina* and *Conotubus*; others only partly closed, open, or undetermined	Yes	No	Yes	Yes	Some (examples in serpulids, sabellids, siboglinids, alvinellids)
	Tube taper	Yes	Basal-most taper (ceriantharians), otherwise constant	No	No	Basal-most taper, otherwise constant	Some taper, some with parallel walls (no taper), and some U-shaped
	Substrate attachment structures	No	Variable (No for ceriantharians; Some in hexacorallians)	No	No	No	Variable; depends on Family (examples in serpulids, sabellids, nereids, terebellids)
Reproduction	Capable of asexual reproduction	Reported in *Cloudina*, *Multiconotubus*, and *Feiyanella*	Yes	Yes	Yes	Yes	Yes
	Equal diameter of daughter tubes	Yes	Not always	Yes	Yes	Yes	Yes
Gut structure		Straight, one-way through-gut	Two-way, sac-like gastrovascular cavity	U-shaped, one-way gut	Straight, one-way through-gut	U-shaped, one-way gut	Straight, one-way through-gut

Supplementary References

1 Hua, H., Chen, Z., Yuan, X., Zhang, L. & Xiao, S. Skeletogenesis and asexual reproduction in the earliest biomineralizing animal *Cloudina*. *Geology* **33**, 277–280, doi:10.1130/G21198.1 (2005).

2 Hou, X.-g. *et al.* An early Cambrian hemichordate zooid. *Current Biology* **21**, 612–616, doi:10.1016/j.cub.2011.03.005 (2011).

3 Stach, T., Gruhl, A. & Kaul-Strehlow, S. The central and peripheral nervous system of Cephalodiscus gracilis (Pterobranchia, Deuterostomia). *Zoomorphology* **131**, 11–24, doi:10.1007/s00435-011-0144-x (2012).

4 Caron, J.-B., Morris, S. C. & Cameron, C. B. Tubicolous enteropneusts from the Cambrian period. *Nature* **495**, 503, doi:10.1038/nature12017 (2013).

5 Nanglu, K., Caron, J.-B., Morris, S. C. & Cameron, C. B. Cambrian suspension-feeding tubicolous hemichordates. *BMC biology* **14**, 56, doi:10.1186/s12915-016-0271-4 (2016).

6 Balthasar, U. & Butterfield, N. J. Early Cambrian “soft-shelled” brachiopods as possible stem-group phoronids. *Acta Palaeontologica Polonica* **54**, 307–315, doi:10.4202/app.2008.0042 (2009).

7 Skovsted, C. B., Brock, G. A., Paterson, J. R., Holmer, L. E. & Budd, G. E. The scleritome of *Eccentrotheca* from the Lower Cambrian of South Australia: Lophophorate affinities and implications for tommotiid phylogeny. *Geology* **36**, 171–174, doi:10.1130/G24385A.1 (2008).

8 Taylor, P. D., Vinn, O. & Wilson, M. A. Evolution of biomineralisation in ‘lophophorates’. *Special Papers in Palaeontology* **84**, 317–333, doi:10.1111/j.1475-4983.2010.00985.x (2010).

9 Hua, H., Chen, Z. & Yuan, X. The advent of mineralized skeletons in Neoproterozoic Metazoa: new fossil evidence from the Gaojiashan Fauna. *Geological Journal* **42**, 263–279, doi:10.1002/gj.1077 (2007).

10 Cai, Y., Schiffbauer, J. D., Hua, H. & Xiao, S. Morphology and paleoecology of the late Ediacaran tubular fossil *Conotubus hemiannulatus* from the Gaojiashan Lagerstätte of southern Shaanxi Province, South China. *Precambrian Research* **191**, 46–57, doi:10.1016/j.precamres.2011.09.002 (2011).

11 Zhuravlev, A. Y., Liñán, E., Vintaned, J. A. G., Debrenne, F. & Fedorov, A. B. New finds of skeletal fossils in the terminal Neoproterozoic of the Siberian Platform and Spain. *Acta Palaeontologica Polonica* **57**, 205–225, doi:10.4202/app.2010.0074 (2012).

12 Budd, G. E. & Jackson, I. S. C. Ecological innovations in the Cambrian and the origins of the crown group phyla. *Philosophical Transactions of the Royal Society B* **371**, 20150287, doi:10.1098/rstb.2015.0287 (2016).

13 Zhang, Z., Holmer, L. E., Ou, Q., Han, J. & Shu, D. The exceptionally preserved Early Cambrian stem rhynchonelliform brachiopod *Longtancunella* and its implications. *Lethaia* **44**, 490–495, doi:10.1111/j.1502-3931.2011.00261.x (2011).

14 Butterfield, N. J. Exceptional fossil preservation and the Cambrian explosion. *Integrative and Comparative Biology* **43**, 166–177, doi:10.1093/icb/43.1.166 (2003).

15 Skovsted, C. B. & Peel, J. S. *Hyolithellus* in life position from the lower Cambrian of North Greenland. *Journal of paleontology* **85**, 37–47, doi:10.1666/10-065.1 (2011).

16 Weigert, A. & Bleidorn, C. Current status of annelid phylogeny. *Organisms Diversity & Evolution* **16**, 345–362, doi:10.1007/s13127-016-0265-7 (2016).
