# Supplementary material for: Discovery of bilaterian-type through-guts in cloudinomorphs from the terminal Ediacaran Period
Source: Nat Commun. 2020 Jan 10;11:205. doi: 10.1038/s41467-019-13882-z (PMC6954273; doi:10.1038/s41467-019-13882-z)
Supplement: Supplementary file 3 — Reporting Summary [file 41467_2019_13882_MOESM3_ESM.pdf]

nature research

Corresponding author(s): James D Schiffbauer and Tara Selly

Last updated by author(s): Nov 18, 2019

Reporting Summary

Nature Research wishes to improve the reproducibility of the work that we publish. This form provides structure for consistency and transparency in reporting. For further information on Nature Research policies, see Authors & Referees and the Editorial Policy Checklist.

Statistics

For all statistical analyses, confirm that the following items are present in the figure legend, table legend, main text, or Methods section.

n/a	Confirmed	
☐	☒	The exact sample size (n) for each experimental group/condition, given as a discrete number and unit of measurement
☒	☐	A statement on whether measurements were taken from distinct samples or whether the same sample was measured repeatedly
☒	☐	The statistical test(s) used AND whether they are one- or two-sided<br>*Only common tests should be described solely by name; describe more complex techniques in the Methods section.*
☒	☐	A description of all covariates tested
☒	☐	A description of any assumptions or corrections, such as tests of normality and adjustment for multiple comparisons
☒	☐	A full description of the statistical parameters including central tendency (e.g. means) or other basic estimates (e.g. regression coefficient) AND variation (e.g. standard deviation) or associated estimates of uncertainty (e.g. confidence intervals)
☒	☐	For null hypothesis testing, the test statistic (e.g. F, t, r) with confidence intervals, effect sizes, degrees of freedom and P value noted<br>*Give P values as exact values whenever suitable.*
☒	☐	For Bayesian analysis, information on the choice of priors and Markov chain Monte Carlo settings
☒	☐	For hierarchical and complex designs, identification of the appropriate level for tests and full reporting of outcomes
☒	☐	Estimates of effect sizes (e.g. Cohen's d, Pearson's r), indicating how they were calculated

Our web collection on statistics for biologists contains articles on many of the points above.

Software and code

Policy information about availability of computer code

Data collection	Primary data collection was conducted via the Scout and Scan Control System 12.0.8059.19531 software, provided by Zeiss Xradia. No custom codes were used.
Data analysis	Data visualization was conducted using TXM3DViewer 1.2.9 (Zeiss Xradia) and segmentation was conducted using AVIZO 9.7.0 (ThermoScientific). No custom codes were used.

For manuscripts utilizing custom algorithms or software that are central to the research but not yet described in published literature, software must be made available to editors/reviewers. We strongly encourage code deposition in a community repository (e.g. GitHub). See the Nature Research guidelines for submitting code & software for further information.

Data

Policy information about availability of data

All manuscripts must include a data availability statement. This statement should provide the following information, where applicable:

- Accession codes, unique identifiers, or web links for publicly available datasets
- A list of figures that have associated raw data
- A description of any restrictions on data availability

This manuscript contains raw and processed data in all figures, with the exception of figures 1, 7, and 8. An availability of data statement is included.

Field-specific reporting

Please select the one below that is the best fit for your research. If you are not sure, read the appropriate sections before making your selection.

☐ Life sciences ☐ Behavioural & social sciences ☒ Ecological, evolutionary & environmental sciences

Ecological, evolutionary & environmental sciences study design

All studies must disclose on these points even when the disclosure is negative.

Study description	This study reports x-ray tomographic data and reconstructions of pyritized cloudinomorph fossils from 550-545 million year old strata of the Montgomery Mountains, Nye County, Nevada, USA. We specifically analyzed 35 fossils, and report potential soft-tissues from 4 of those.
Research sample	The samples analyzed are 550-545 Ma cloudinomorph fossils from the Montgomery Mountains, Nye County, Nevada, USA. We chose these samples for several reasons: (1) this locality and fossils were recently described in a Smith et al. Proc. Roy. Soc. B manuscript as a new suite of terminal Ediacaran tubular organisms; (2) they illustrate three-dimensional preservation, but in many cases appeared largely hollow, thus increasing their potential for capturing internally preserved features or tube-wall structures; and (3) cloudinomorphs of this sort have recently been a focus of renewed attention, both because of their presence during the decline of the Ediacara biota and their potential utility in pending subdivisions of the Ediacaran Period.
Sampling strategy	From the previous Smith et al. collection, 35 fossil samples were chosen based on their apparent three-dimensionality in preservation. While more than 35 samples exist in the collection, we targeted those that we felt had the highest potential for internal preservation.
Data collection	Data collection was conducted at the University of Missouri X-ray Microanalysis Core Facility using a Zeiss Xradia 510 Versa x-ray microscope and a Zeiss Sigma 500 VP scanning electron microscope. Serial x-ray attenuation slices were viewed and segmented using Avizo 9.7 software (ThermoScientific). Primary data collection efforts were conducted by Selly and Schiffbauer; Jacquet performed primary data visualization.
Timing and spatial scale	Field-based sampling was conducted primarily by coauthors Smith and Nelson in 2017. Data collection using the aforementioned methods took place between 03/06/18 to 08/27/18. As both instruments utilized are housed within a for-service University core facility, scans were conducted off peak hours of operation. Data were collected at micrometric to millimetric scale.
Data exclusions	Specimens figured are those that show soft-tissue preservation, whereas the remaining 31 samples are not figured due to their lack of soft tissues. Otherwise, no data are excluded from our analyses.
Reproducibility	Scan parameterization is reported in-text. Samples will be reposited at the Smithsonian Institution, Washington D.C. except for the single sample that was destructively prepared for electron microscopy.
Randomization	This is not relevant to our study. Samples were collected from a fossiliferous horizon based on field observation and only those with the highest potential were chosen for analysis.
Blinding	Blinding is not relevant to this study as there were no participants.

Did the study involve field work? ☒ Yes ☐ No

Field work, collection and transport

Field conditions	Samples were predominantly collected in early 2017 in Nevada, where the average temperature was ~60°F. Rainfall was minimal.
Location	Sampling site is located near the Johnnie Townsite, approximately 36 22' 30"N 116 07' 30"W.
Access and import/export	Samples were collected under permit to coauthor Smith by the Bureau of Land Management in Nevada, Permit N-94103, with collection dates from 01/01/2017 to 12/31/2017.
Disturbance	No disturbance was caused during collection.

Reporting for specific materials, systems and methods

We require information from authors about some types of materials, experimental systems and methods used in many studies. Here, indicate whether each material, system or method listed is relevant to your study. If you are not sure if a list item applies to your research, read the appropriate section before selecting a response.

Materials & experimental systems

n/a	Involved in the study
☒	☐ Antibodies
☒	☐ Eukaryotic cell lines
☐	☒ Palaeontology
☒	☐ Animals and other organisms
☒	☐ Human research participants
☒	☐ Clinical data

Methods

n/a	Involved in the study
☒	☐ ChIP-seq
☒	☐ Flow cytometry
☒	☐ MRI-based neuroimaging

Palaeontology

Specimen provenance

Samples were collected under permit to coauthor Smith by the Bureau of Land Management in Nevada, Permit N-94103, with collection dates from 01/01/2017 to 12/31/2017. Sampling site is located near the Johnnie Townsite, approximately 36 22' 30"N 116 07' 30"W.

Specimen deposition

Samples will be reposited in the Smithsonian Institution, Washington D.C.

Dating methods

No new dates are provided.

☐ Tick this box to confirm that the raw and calibrated dates are available in the paper or in Supplementary Information.
