# Supplementary material for: Discovery of bilaterian-type through-guts in cloudinomorphs from the terminal Ediacaran Period
Source: Nat Commun. 2020 Jan 10;11:205. doi: 10.1038/s41467-019-13882-z (PMC6954273; doi:10.1038/s41467-019-13882-z)
Supplement: Supplementary file 4 — Description of Additional Supplementary Files [file 41467_2019_13882_MOESM4_ESM.pdf]

Description of Additional Supplementary Files

File Name: Supplementary Movie 1
Description: 3D reconstruction full movie of sample illustrated in Figs. 4, 5, and 6. Movie progression: (1) sequential 2D tomograph slices moving through the host rock and fossil, bright regions indicate pyrite tube wall and gut. Direction proceeds from the lower part of Fig. 5F towards the upper part. (2) Layered dissolve of host rock, revealing Avizo-segmented data. Red color = tube wall and disseminated pyrite. (3) Removal of disseminated and non-continuous pyrite around the tube wall structure. (4) Rotation and reveal of gut structure (gold/orange). Hints of tube wall funnel/transverse annulation structure can be observed during rotation. (5) Dissolution of tube wall for full gut reveal.

File Name: Supplementary Movie 2
Description: 2D stacked tomograph, latitudinal cross-section slices through sample illustrated in Figs. 4, 5, and 6. Movie progresses from upper part of Fig. 5f to lower part (reverse from Supplementary Movie 1).

File Name: Supplementary Movie 3
Description: 2D stacked tomograph, longitudinal cross-section slices through sample illustrated in Figs. 4, 5, and 6. Sample in the reverse orientation (180°) as that from Fig. 5f.
